# Hyperthermia intravesical chemotherapy acts as a promising alternative to bacillus Calmette–Guérin instillation in non-muscle-invasive bladder cancer: a network meta-analysis

**DOI:** 10.3389/fonc.2023.1164932

**Published:** 2023-05-12

**Authors:** Na Zeng, Meng-Yao Xu, Jian-Xuan Sun, Chen-Qian Liu, Jin-Zhou Xu, Ye An, Xing-Yu Zhong, Si-Yang Ma, Hao-Dong He, Qi-Dong Xia, Shao-Gang Wang

**Affiliations:** Department and Institute of Urology, Tongji Hospital, Tongji Medical College, Huazhong University of Science and Technology, Wuhan, China

**Keywords:** hyperthermia intravesical chemotherapy, HIVEC, BCG, adjuvant therapy, non-muscle-invasive bladder cancer, network meta-analysis

## Abstract

**Introduction:**

With the shortage of bacillus Calmette–Guérin (BCG) vaccine, it is important to find an alternative to BCG instillation, which is the most commonly used adjuvant treatment for non-muscle-invasive bladder cancer (NMIBC) patients after transurethral resection of bladder tumor treatment (TURBt) to delay tumor recurrence. Hyperthermia intravesical chemotherapy (HIVEC) with mitomycin C (MMC) is a potential treatment choice. We aim to compare HIVEC with BCG instillation for the preventive efficacy of bladder tumor recurrence and progression.

**Methods:**

A network meta-analysis (NMA) was taken with MMC instillation and TURBt as the attached comparators. Randomized controlled trials (RCTs) with NIMBC patients after TURBt were included. Articles with pure BCG unresponsive patients and combined therapies were excluded. The study protocol was registered in the International Prospective Register of Systematic Reviews (PROSPERO, CRD42023390363).

**Results:**

It was found that HIVEC had a non-significant 22% relative reduction in bladder tumor recurrence compared with BCG instillation [HIVEC vs. BCG: HR 0.78, 95% credible interval (CrI) 0.55–1.08] and a nonsignificant higher risk of bladder tumor progression (BCG vs. HIVEC: HR 0.77, 95% CrI 0.22–3.03).

**Discussion:**

HIVEC is a potential alternative to BCG, and it is expected to be the standard therapy for NMIBC patients after TURBt during the global shortage of BCG.

**Systematic Review Registration:**

PROSPERO identifier, CRD42023390363

## Introduction

Bladder cancer (BC) is the 10th most commonly diagnosed cancer worldwide, with an age-standardized incidence rate (per 100,000 person-years) of 9.5 for men and 2.4 for women worldwide (1). Based on the report in 2016 (2), roughly 75% of bladder cancer patients have non-muscle invasive bladder cancer (NMIBC), with high 5-year rates of NMIBC recurrence, ranging from 50% to 70%, and alarming 5-year rates of progression, ranging from 10% to 30%, which appeals for more effective treatments for NMIBC patients. Considering that tumors commonly recur and can even progress to muscle-invasive bladder cancer (MIBC) after transurethral resection of bladder tumor treatment (TURBt) (1), which is the conventional treatment of bladder cancer, TURBt supplemented with postoperative adjuvant therapies is recommended as the standard treatment for NMIBC.

To date, postoperative adjuvant therapies consist of intravesical bacillus Calmette–Guérin (BCG) immunotherapy and intravesical chemotherapy, including mitomycin C (MMC) instillation, epirubicin instillation, and pirarubicin instillation. Nevertheless, BCG after TURBt is recognized to be superior to TURBt plus chemotherapy for preventing the recurrence of NMIBC, while no statistically significant difference is confirmed between MMC and BCG for progression, survival, and cause of death (1), which indicates that BCG may be the most effective treatment. Even so, the limitations of BCG therapy, such as higher toxicity risk and severe side effects compared with MMC, recent worldwide shortages of BCG vaccines (3), and BCG unresponsive category (2), which includes BCG-refractory disease and a subset of the patients with relapsing BCG who have a recurrence within 6 months of last exposure to BCG, should not be ignored. New drugs and strategies are required.

In 2016, Arends et al. (4) discussed a new adjuvant treatment strategy: treat patients with MMC perfusion combined with local hyperthermia at 42 ± 2°C with the help of hyperthermia device, which is now defined as hyperthermia intravesical chemotherapy (HIVEC) with MMC. The trial performed no statistical significance between HIVEC and BCG in recurrence-free time, suggesting that HIVEC is a safe and effective treatment option as a possible alternative to BCG in patients with intermediate- and high-risk papillary NMIBC, especially given the recent worldwide shortages of BCG. Meanwhile, one meta-analysis (5) has evaluated the individual data from 327 patients enrolled in four RCTs that have compared HIVEC vs. BCG, reaching a similar conclusion after taking account of HIVEC having equivalent oncological outcomes and a similar safety profile when compared to BCG maintenance therapy for patients with intermediate- and high-risk NMIBC. However, the result should be treated regarding the limited sample size of this meta-analysis. Here, we aim to compare HIVEC with BCG instillation in the preventive efficacy of bladder tumor recurrence and progression by a network meta-analysis (NMA), synthesizing the comparison between HIVEC/BCG and MMC instillation.

## Materials and methods

### Search strategy and selection criteria

PubMed (Medline), Ovid (Embase), and Cochrane Library were searched to screen available randomized controlled trials (RCTs) followed by the Preferred Reporting Items for Systematic Reviews and Meta-analyses (PRISMA) guidelines (6) from the time of in-caption to 9 January 2023. The PRISMA checklist describes the process of including documents, the number of excluded documents at each step, and the reasons for exclusion. Since the pre-search showed that HIVEC was commonly combined with MMC, and no RCTs focus on the comparison between HIVEC and TURBt, the strategy of article searching consists of two parts: (i) HIVEC versus BCG or MMC perfusion and (ii) BCG perfusion versus MMC perfusion versus TURBt. Search terms and results are provided in **File S1**.

For all studies, patients were pathologically diagnosed as non-muscle-invasive or superficial or Ta/T1 with/without Tis bladder cancer. Only RCTs evaluating the effectiveness of treatments (HIVEC, BCG, and MMC) for NMIBC after TURBt are selected. Bladder cancer recurrence or progression (to T2 or greater) should be assessed based on at least cystoscopy and urine cytology. Eligible studies excluded study groups with purely BCG unresponsive patients or those subjected to combined therapy like BCG plus MMC instillation. Studies with a lack of usable data were excluded either. The study protocol was registered in the International Prospective Register of Systematic Reviews (PROSPERO, CRD42023390363).

### Data extraction and quality assessment

Data to a pre-designed data extraction form were extracted, including the name of the first author, publication year, country, study center, the number of eligible and analyzed patients, participant inclusion and exclusion criteria, baseline age, sex portion, tumor characteristics (primary/recurrent, risk of recurrence, pT stage, grade, and carcinoma *in situ*), description of intervention and comparator, and outcome. The targeted outcomes are recurrence-free survival (RFS) and progression-free survival (PFS), and disease-free survival (DFS) is regarded the same as RFS. For the above time-to-event data, we extracted the natural logarithm of the hazard ratio (HR) and its standard error from trial reports. The intention-to-treat analysis is the first choice if possible, or the per-protocol analysis will be extracted. Where they were not explicitly reported, we estimated them from data extracted from Kaplan–Meier plots or provided by authors where possible based on the study of Woods et al. (7).

Two reviewers, Z.N. and X.M.Y., independently assessed the risk of bias using Cochrane’s risk-of-bias tool for randomized trials (RoB2) and the criteria specified in the Cochrane Handbook for Systematic Reviews of Interventions (8). Differences were resolved by discussion. This included assessment of random sequence generation and allocation concealment, blinding of participants and doctors, blinding of outcome assessors, outcome measurement (more than 10% missing data were considered high risk), and selective reporting of outcomes.

### Data analysis

In network meta-analysis, the node-splitting model was chosen to check the consistency. We assessed the presence of statistical heterogeneity within the pairwise comparisons using the *I*² statistic, the percentage of variability that cannot be attributed to random error. Considering the heterogeneity, a Bayesian consistency network model was generated under a random-effects model with the “gemtc” package in R v4.1.1 to evaluate the comprehensive HR between HIVEC and BCG in tumor recurrence and progression. Additionally, subgroup analysis of RFS with regard to male percentage, median follow-up, median age, region, and whether the carcinoma *in situ* patients were excluded or not was taken.

## Results

### Descriptive characteristics of studies included

**Figure 1:**
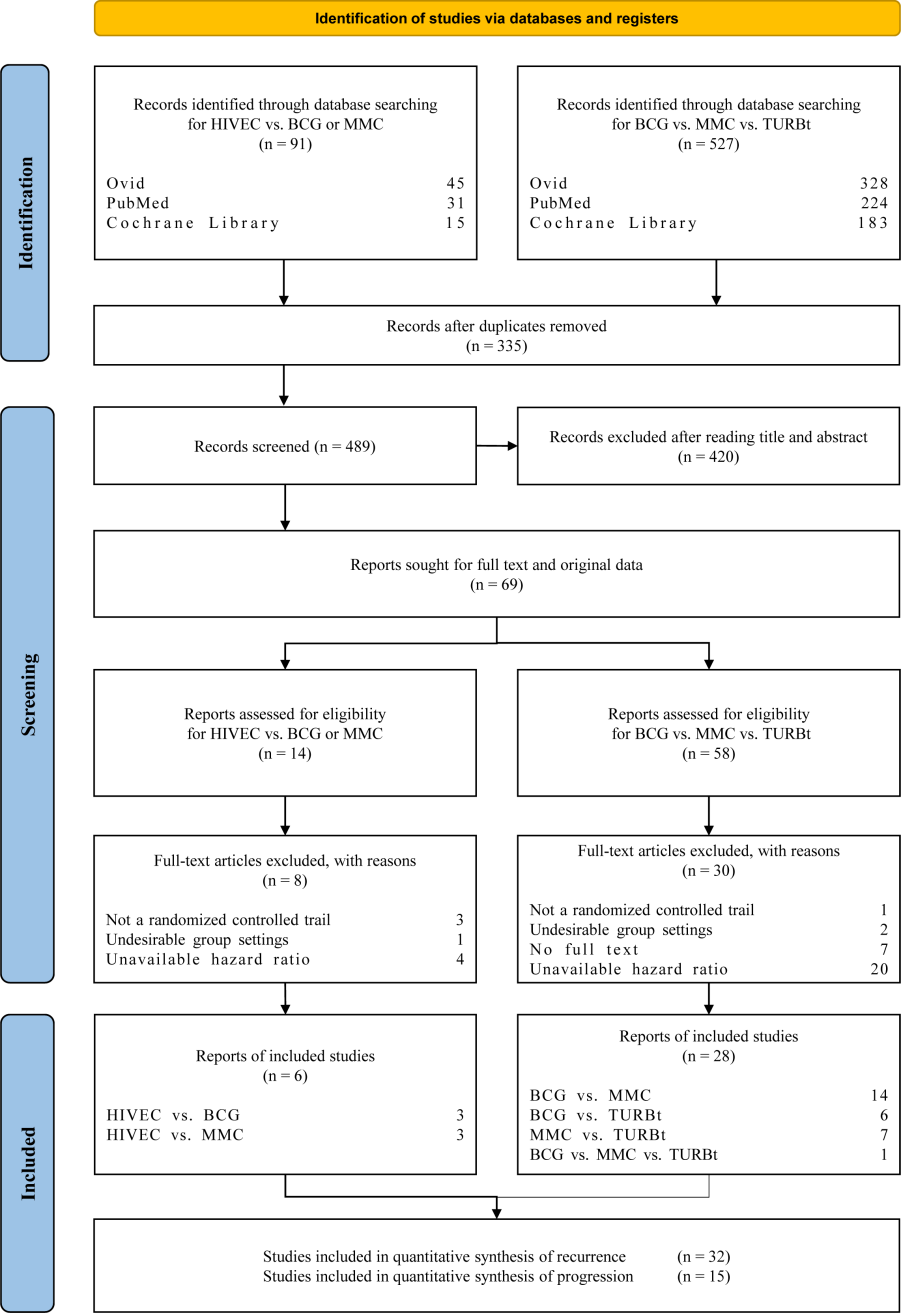
PRISMA (Preferred Reporting Items for Systematic Reviews and Meta-Analyses) flowchart for study selection of the RCTs related to hyperthermia intravesical chemo-therapy (HIVEC), bacillus Calmette–Guérin (BCG), mitomycin C (MMC), and transurethral resection of bladder tumor treatment (TURBt).

We assessed 72 articles by title and abstract review (**Figure 1**) from 826 records initially identified. The exact reasons for excluding 38 articles are summarized in **File S2**. A total of 34 trials were finally included in the analyses, and 32 trials of recurrence and 15 trials of progression were analyzed, respectively. From 1980 to 2022, 36 articles were published in English, and the remaining 2 articles were written in German and Japanese, respectively, which were read with the help of translation software. Characteristics of patients and outcomes are summarized in **Table 1**. At a median age of 59–76.5 years, the median follow-up of trials ranged from 12 months to more than 10 years. A total of 75 arms with 7,107 enrolled participants are included in the quantitative synthesis. Among the 75 arms, 7 arms receive HIVEC, 27 arms receive BCG, 26 arms receive MMC, and 15 arms do not receive any treatment after TURBt.

**Table 1:** Summary of RCTs included in the network meta-analysis.

Study	Published Year	Intervention vs. Comparator (*n*)	Male Percentage (%)	Median Age (years)	Median Follow-up	HR of RFS	HR of PFS
Arends (4)	2016	HIVEC vs. BCG (68 vs. 74)	83.7	66.3	25.6 mo	0.46 (0.20–1.09)	NA
Tan (9)	2019	HIVEC vs. BCG (48 vs. 56)	75.0	76.5	24 mo	1.33 (0.84–2.10)	NA
Guerrero Ramos (10)	2022	HIVEC vs. BCG (25 vs. 25)	86.0	73.6	33.7 mo	0.48 (0.11–2.03)	0.16 (0.02–1.40)
Colombo (11)	2010	HIVEC vs. MMC (35 vs. 40)	83.3	NA	>10 yr	0.33 (0.17–0.64)	NA
Tan (12)	2022	HIVEC vs. MMC (75 vs. 111)	70.5	69.5	24 mo	0.75 (0.46–1.22)	2.87 (0.83–9.98)
Angulo (13)	2022	HIVEC1 vs. MMC (107 vs. 106)	83	67.5	23 mo	0.77 (0.43–1.40)	NA
		HIVEC2 vs. MMC (106 vs. 106)	82	67	23 mo	0.78 (0.43–1.40)	NA
Smits (14)	1998	BCG vs. MMC (81 vs. 43)	NA	NA	3 yr	1.33 (0.77–2.27)	1.50 (0.61–3.67)
Lundholm (15)	1996	BCG vs. MMC (125 vs. 125)	84	68	39 mo	0.70 (0.50–0.97)	NA
Witjes (16)	1993	BCG1 vs. MMC (117 vs. 136)	83.3	65.7	4.5 yr	1.41 (0.93–2.15)	NA
		BCG2 vs. MMC (134 vs. 136)	83.5	65.8	4.5 yr	1.22 (0.77–1.91)	NA
Herr (17)	1995	BCG vs. TURBt (43 vs. 43)	75.6	60.5	124 mo	0.47 (0.18–1.28)	0.42 (0.16–1.11)
Herr (18)	1997	BCG vs. TURBt (25 vs. 23)	66.7	59	15 yr	NA	0.71 (0.26–1.99)
Lamm (19)	1980	BCG vs. TURBt (18 vs. 19)	94.6	64.2	<12 mo	0.77 (0.13–4.68)	NA
Rintala (20)	1991	MMC vs. BCG (58 vs. 51)	73.4	67.5	24 mo	2.30 (1.35–3.91)	NA
Witjes (21)	1998	BCG vs. MMC (159 vs. 168)	81.5	NA	>10 yr	0.86 (0.63–1.19)	0.56 (0.28–1.11)
Friedrich (22)	2007	BCG vs. MMC (163 vs. 179)	81	67	2.9 yr	1.01 (0.66–1.54)	NA
Gårdmark (23)	2007	BCG vs. MMC (125 vs. 125)	84	68	123 mo	NA	0.75 (0.42–1.36)
Ojea (24)	2007	MMC vs. BCG1 (149 vs. 142)	87.6	64.3	54.9 mo	1.74 (1.15–2.62)	0.98 (0.47–2.06)
		BCG2 vs. MMC (139 vs. 149)	86.4	64.2	56.8 mo	0.84 (0.69–1.03)	1.02 (0.49–2.14)
Isbarn (25)	2008	BCG vs. MMC (163 vs. 179)	81	67	2.9 yr	1.09 (0.71–1.68)	NA
Niigima (26)	1983	MMC vs. TURBt (139 vs.139)	NA	NA	12 mo	0.73 (0.5–1.05)	NA
Tsushima (27)	1987	MMC vs. TURBt (37 vs. 33)	81.4	66.1	16 mo	0.36 (0.18–0.73)	NA
Yamakomo (28)	1990	BCG vs. TURBt (23 vs. 21)	72.7	62.5	36 mo	0.31 (0.10–0.96)	NA
Skrege (29)	1996	BCG vs. TURBt (102 vs. 122)	77.7	65.6	20.2 mo	0.62 (0.39–0.99)	NA
		MMC vs. TURBt (113 vs. 122)	79.6	66.1	20.2 mo	0.51 (0.49–1.40)	NA
		BCG vs. MMC (102 vs. 113)	82.3	64.3	20.2 mo	0.96 (0.61–1.51)	NA
Sakamoto (30)	2001	MMC vs. TURBt (13 vs. 12)	64	69.4	45 mo	0.48 (0.05–5.02)	NA
De Nunzio (31)	2011	MMC vs. TURBt (97 vs. 105)	65.8	61	90 mo	0.20 (0.10–0.40)	NA
Akaza (32)	1987	MMC vs. TURBt (150 vs. 148)	81.5	62.1	24 mo	0.70 (0.50–0.96)	NA
		MMC vs. TURBt (139 vs.139)	74.5	62.9	24 mo	0.71 (0.49–1.01)	NA
Kim (33)	1989	MMC vs. TURBt (21 vs. 22)	88.4	54.4	31.3 mo	1.07 (1.78–0.64)	0.52 (0.10–2.62)
Pagano (34)	1991	BCG vs. TURBt (70 vs. 63)	NA	NA	21.3 mo	0.34 (0.22–0.50)	0.25 (0.07–0.85)
Melekos (35)	1993	BCG vs. TURBt (62 vs. 32)	83	67.3	25.4 mo	0.39 (0.20–0.75)	0.29 (0.09–0.95)
Vegt (36)	1995	BCG1 vs. MMC (140 vs. 148)	NA	NA	>3 yr	1.57 (1.11–2.21)	1.02 (0.38–2.72)
		BCG2 vs. MMC (149 vs. 148)	NA	NA	>3 yr	1.12 (0.78–1.60)	1.01 (0.39–2.63)
Lamm (37)	1995	BCG vs. MMC (191 vs. 186)	83	67	33.1 mo	0.70 (0.52–0.94)	0.80 (0.43–1.50)
Tolley (38)	1996	MMC1 vs. TURBt (146 vs. 157)	NA	63.3	>3 yr	0.50 (0.36–0.70)	0.82 (0.4–1.68)
		MMC2 vs. TURBt (149 vs. 157)	NA	NA	NA	0.66 (0.48–0.91)	0.84 (0.42–1.52)
Malmstrom (39)	1999	BCG vs. MMC (125 vs. 125)	NA	NA	5 yr	0.71 (0.52–0.98)	NA
Mangiarotti (40)	2008	BCG vs. MMC (46 vs. 46)	72.83	65.7	>3 yr	0.84 (0.47–1.49)	NA
Järvinen (41)	2009	BCG vs. MMC (44 vs. 45)	72	67.5	>10 yr	0.49 (0.30–0.81)	0.41 (0.14–1.23)

HIVEC, hyperthermia intravesical chemotherapy with mitomycin C; BCG, bacillus Calmette–Guérin perfusion; MMC, mitomycin C perfusion; HR, hazard ratio; RFS, recurrence-free survival; PFS, progression-free survival; mo, months; yr, years.

The quality assessment figures and graph using RoB2 are performed in **Figure S1**. Three articles performed a certain risk of bias deriving from intended intentions, which is unavoidable because of the different procedures of the four arms. Since there is no flow of patients between the intervention arm and comparator arm after randomization in the remaining 31 articles, we felt low risk of bias in the part of derivation from the intended intentions.

### Recurrence-free survival and progression-free survival analysis

**Figure 2:**
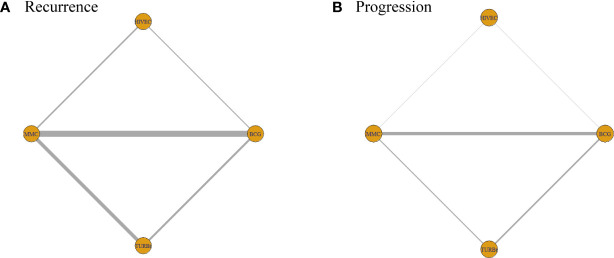
Network diagrams of network comparisons on **(A)** recurrence and **(B)** progression.

**Figure 3:**
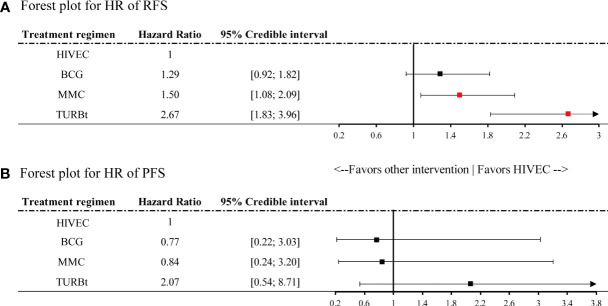
Forest plot for hazard ratio of **(A)** recurrence-free survival (RFS) and **(B)** progression-free survival (PFS). Red points refer to a significant result and black points refer to a nonsignificant result.

**Figure 4:**
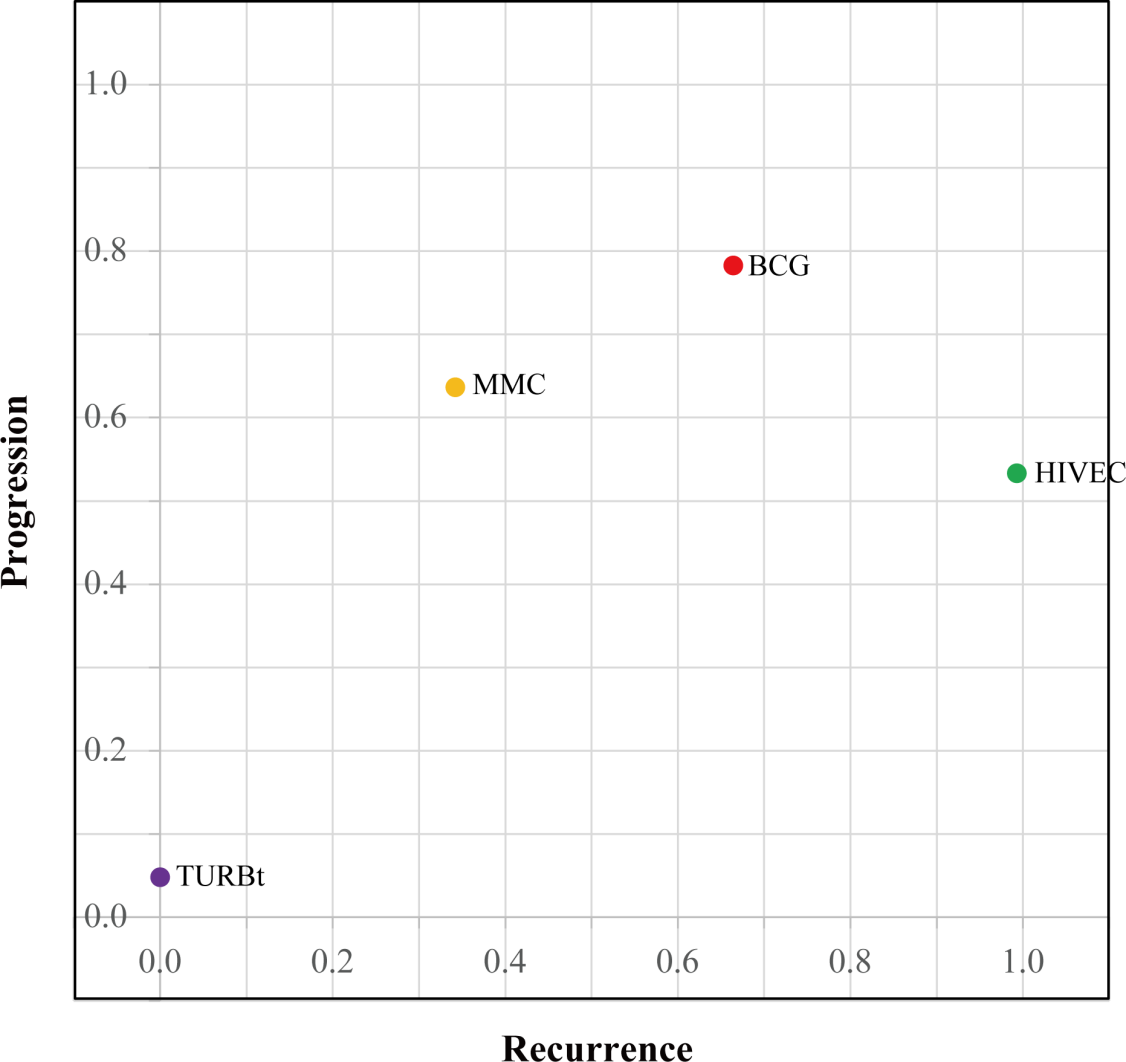
SUCRA plot for ranking recurrence and progression prevention.

The network diagrams of net comparisons are presented in **Figure 2**. The forest plot performed the relative effects of four treatment groups on HR of RFS (**Figure 3A**) and PFS (**Figure 3B**), with HIVEC as the reference. Efficacy estimates of recurrence and progression outcomes calculated from NMA are listed in **Table 2**, expressing the effect sizes in recurrence below the diagonal and progression above the diagonal. Posterior ranking probabilities of four treatment strategies for recurrence and progression were calculated using a Bayesian random-effects hierarchical model (**Figure S2**), and the SUCRA rank is drawn in **Figure 4**.

**Table 2:** Efficacy estimate table from network meta-analysis with 95% credible intervals.

**HIVEC**	1.30 (0.33, 4.62)	1.19 (0.31, 4.16)	0.48 (0.11, 1.84)
0.78 (0.55, 1.08)	**BCG**	0.91 (0.64, 1.35)	0.37 (0.22, 0.64)
0.67 (0.48, 0.92)	0.86 (0.74, 0.99)	**MMC**	0.41 (0.23, 0.70)
0.37 (0.25, 0.55)	0.48 (0.38, 0.61)	0.56 (0.45, 0.69)	**TURBt**

A significant reduction in the risk of recurrence of NMIBC is shown for HIVEC compared with MMC perfusion and TURBt [MMC vs. HIVEC: HR 1.50, 95% credible interval (CrI) 1.08–2.09; TURBt vs. HIVEC: HR 2.67, 95% CrI 1.83–3.96], with nonsignificant 22% relative reduction compared with BCG perfusion (HIVEC vs. BCG: HR 0.78, 95% CrI 0.55–1.08). Concerning tumor progression, however, BCG and MMC perfusion seems to be more effective than HIVEC in the NMIBC progression, but no significant differences exist between HIVEC and the other three arms (BCG vs. HIVEC: HR 0.77, 95% CrI 0.22–3.03; MMC vs. HIVEC: HR 0.84, 95% CrI 0.24–3.20; TURBt vs. HIVEC: HR 2.07, 95% CrI 0.54–8.71). According to the SUCRA plot for ranking, HIVEC is the most effective therapy considering recurrence outcomes, followed by BCG, MMC, and TURBt. The rank for the prevention of tumor progression is BCG, MMC, HIVEC, and TURBt.

Results of consistency check and heterogeneity test are provided in **Figure S3** and **Figure S4**. For relative hazard ratio related to HIVEC and other arms, good consistency is shown for tumor recurrence, but poor consistency is shown for tumor progression. Poor heterogeneity was observed for HR of both RFS and PFS; thus, the random-effects model was chosen.

### Subgroup analysis of RFS

**Figure 5:**
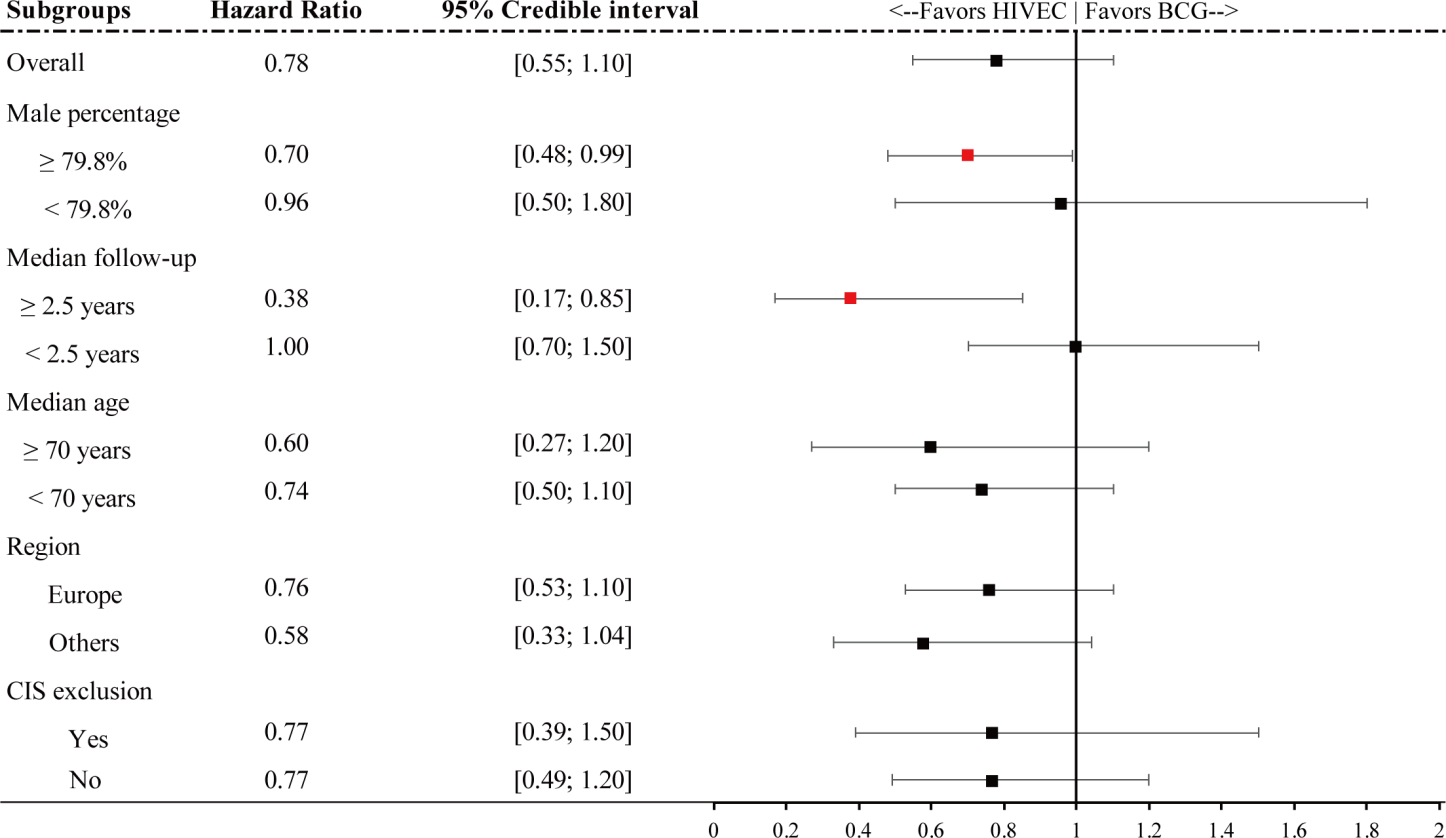
Forest plot for hazard ratio RFS of subgroups. Red points refer to a significant result and black points refer to a nonsignificant result.

Articles included in RFS network meta-analysis were divided into two groups according to the male percentage, median follow-up, median age, region, and whether the carcinoma *in situ* patients were excluded or not, respectively. Since studies included in the quantitative synthesis of progression are inadequate to divide into two groups, only the relative effectiveness of RFS is calculated, shown in **Figure 5**. Based on the fact that the age-standardized incidence rate (per 100, 000 person-years) is 9.5 for men and 2.4 for women worldwide, we simply regarded 79.8% as the watershed. If the male percentage of one article is greater than 79.8%, it may indicate a higher portion of male NMIBC patients included. Similarly, one article with a median age of included patients greater than 70 years will be regarded as a higher portion for high-risk NMIBC patients, given that age (≥70 years) is a risk factor of high-risk NMIBC (1). Meanwhile, considering the importance of the 5-year survival rate of NMIBC, 2.5 years was chosen as the cutoff point for the median follow-up. By the way, articles without adequate information to be classified are included in both subgroups to obtain a more conservative result.

It is shown that for a higher portion of male patients, HIVEC plays a significant 30% relative reduction compared with BCG perfusion (HR 0.70, 95% CrI 0.48–0.99). In addition, for articles with longer follow-up (median follow-up ≥ 2.5 years), HIVEC shows significant efficacy in tumor recurrence prevention (HR 0.38, 95% CrI 0.17–0.85). No significant results are performed in the other three subgroup analysis.

## Discussion

Recently, prognostic-related factors of urogenital system tumors have been widely discussed for application in cancer detection and treatment (42–45). Although the survival for NMIBC is favorable, the objectionable rate of bladder tumor recurrence should not be ignored. Numerous factors affect the recurrence and progression rate of bladder cancer, including tumor characteristics (1), organic pollutants (42), and especially postoperative BCG maintenance therapy. It is acknowledged that intravesical BCG instillation is regarded to play a positive role in preventing the recurrence of intermediate- and high-risk NMIBC bladder tumor recurrence. However, the limitations of BCG therapy such as high toxicity risk and severe side effects appeal to better adjuvant treatments for NMIBC after TURBt.

In our network meta-analysis, HIVEC, the emerging adjuvant treatment, non-significantly performs better than BCG perfusion for preventing NMIBC recurrence, while showing worse efficacy when it comes to the prevention of tumor progression. In the subgroup analysis of male percentage and median follow-up, a significantly high RFS for HIVEC compared to BCG perfusion was observed, which likely suggested that HIVEC benefited male patients with long-term efficacy regarding tumor recurrent prevention. Otherwise, no differences exist between CIS exclusion groups and CIS inclusion groups, though Tan et al. (9) found that HIVEC benefited non-CIS NMIBC patients but was unfavorable for patients with CIS at baseline compared to BCG perfusion.

To sum up, the advantage in recurrence prophylaxis and the inhibition effect compared to TURBt on tumor progression support HIVEC as a substitute for BCG perfusion or even as a priority choice for NMIBC patients, especially for the current shortage of BCG vaccine. Two RCTs (9, 46) supported that the overall rate and grade of study therapy-related AEs were similar between the two treatments, and a retrospective study (47) from 2016 to 2017 proposed that HIVEC has a more favorable AE profile compared with BCG. Additionally, a prospective observational study (48) from 2017 to 2020 clearly expressed that the BCG group had a significantly higher incidence of adverse effects than the HIVEC group (*p* = 0.003). It also held that HIVEC with mitomycin therapy scored better in terms of tolerance and cost benefit, considering the side effect profile, cost, and time involved with the treatment of moderate and severe adverse effects of BCG.

The credible effect of HIVEC compared to conventional chemotherapy of bladder tumors may hinge on the “heating” operation instead of the choice of the perfusion drugs. Heating not only directly affects tumor cancers but also enhances the permeability of cell membranes, promoting drug penetration into the bladder (49). In the process, heat shock protein is released from cancer cells, activating the adaptive T-cell response (50). This presumed synergistic effect of hyperthermia and chemotherapy was identified *in vitro* for several chemotherapeutic agents, including MMC, epirubicin (EPI), and gemcitabine (GEM) (51). Considering that this NMA focuses on HIVEC with MMC, it puts forward a new perspective: explore the combination of hyperthermia and other chemotherapeutic drugs instead of MMC. It has been found that using MMC or EPI in HIVEC did not influence the response to treatment (*p* = 0.157) (52). For example, one study found that the therapeutic rank of GEM was superior to that of BCG in their network model (53). HIVEC with GEM seems to be worth discussing.

Notably, recent studies inspired a rethink of more specific subtypes of bladder tumors when evaluating the effect of BCG instillation. Variant histology has been verified to influence the prognostic behavior in both NMIBC (54) and MIBC (55). For example, being nested in the high-risk category showed limited response to BCG therapy while BCG was proven as an effective treatment in pT1 squamous NMIBC (54, 56). However, the baseline characteristics of bladder tumor type in all studies we included were provided based on the novel WHO 2022 classification, with a lack of a description of the samples’ histology, which certainly impacted the analysis result. More studies on the effect of HIVEC and BCG therapy for NMIBC with detailed histology analysis are expected. Additionally, we excluded articles that targeted BCG unresponsive patients only when designing the NMA, which helps to reduce a possible negative impact on the evaluation of BCG’s effect. Retrospective studies have shown the value of HIVEC in BCG failure (57). Though BCG is recommended as the most effective first-line intravesical therapy in NMIBC, up to 40%–50% of patients will eventually recur after BCG, with the recommended radical cystectomy, which may, however, represent an overtreatment, especially for those patients with non-high-grade BCG failure (58). Furthermore, Sri et al. (59) found that HIVEC with MMC steered clear of a technically more challenging cystectomy or a compromise on the oncological outcome compared to those patients undergoing cystectomy immediately post-BCG failure. For patients with BCG unresponsive disease, we suggest HIVEC as a feasible treatment.

There are several limitations in the present NMA. The major limitation is the small sample size and an inadequate follow-up of the RCTs between HIVEC and BCG. More well-designed RCTs are required to enhance the reliability of the findings of NMAs. That is why we choose the network meta-analysis rather than the meta-analysis. Moreover, the long year range (1980–2022) of studies that we included should be noted. Although the quality assessment result of studies showed a good performance and ensured the quality of the analysis, the same postoperative adjuvant treatment of NMIBC after TURBt such as BCG instillation in each study were slightly optimized with the medical development, which probably influenced the analysis. The hiatus of the direct comparison between HIVEC and TURBt may also influence the network model, especially in disease progression, as the nonsignificant differences between HIVEC and TURBt showed, which is inconsistent with clinical experience. For another, the heterogeneity between studies regarding progression is generally high. Network meta-regression was performed but failed to reveal any impact of potential factors on efficacy.

## Conclusion

This network meta-analysis shows a better effect of HIVEC compared with BCG therapy on the prevention of bladder tumor recurrence but a worse effect of HIVEC on the prevention of tumor progression, with no statistical significance shown. Subgroup analysis reveals the promising benefit of HIVEC in male patients and long-term efficacy for the RFS of NMIBC. It inspires a new adjuvant treatment for NIMBC after TURBt to prevent bladder tumor recurrence.

## Data availability statement

The original contributions presented in the study are included in the article/**Supplementary Material**. Further inquiries can be directed to the corresponding authors.

## Author contributions

NZ, J-XS, Q-DX, and S-GW contributed to developing the main research question, carrying out the literature search, collecting the included studies’ information, and describing the results. NZ and M-YX performed the meta-analysis and wrote the first draft of the manuscript. X-YZ, S-YM, and H-DH contributed to developing the main research question and revised the manuscript. C-QL, J-XS, and YA revised the manuscript. All authors contributed to the article and approved the submitted version. NZ, Q-DX, and S-GW contributed equally to this study.

## Supplementary material

File S1 search terms and results

File S2 the exact reasons for excluding 38 articles

Supplementary Figure 1 the quality assessment figures and graph using RoB2

Supplementary Figure 2 posterior ranking probabilities of four treatment strategies for recurrence and progression

Supplementary Figure 3 results of consistency check

Supplementary Figure 4 results of heterogeneity test
