# Supplementary material for: Hyperthermia intravesical chemotherapy acts as a promising alternative to bacillus Calmette–Guérin instillation in non-muscle-invasive bladder cancer: a network meta-analysis
Source: Front Oncol. 2023 May 12;13:1164932. doi: 10.3389/fonc.2023.1164932 (PMC10213538; doi:10.3389/fonc.2023.1164932)
Supplement: File S1 — search terms and results [file DataSheet_5.pdf]

Part 1: HIVEC versus BCG or MMC

1.1 Search terms in PubMed

PubMed	Search terms	results
1	((((((((((((((((((Neoplasm, Urinary Bladder[Title/Abstract]) OR (Urinary Bladder Neoplasm[Title/Abstract])) OR (Bladder Tumors[Title/Abstract])) OR (Bladder Tumor[Title/Abstract])) OR (Tumor, Bladder[Title/Abstract])) OR (Tumors, Bladder[Title/Abstract])) OR (Neoplasms, Bladder[Title/Abstract])) OR (Bladder Neoplasms[Title/Abstract])) OR (Bladder Neoplasm[Title/Abstract])) OR (Neoplasm, Bladder[Title/Abstract])) OR (Urinary Bladder Cancer[Title/Abstract])) OR (Cancer, Urinary Bladder[Title/Abstract])) OR (Malignant Tumor of Urinary Bladder[Title/Abstract])) OR (Cancer of the Bladder[Title/Abstract])) OR (Bladder Cancer[Title/Abstract])) OR (Bladder Cancers[Title/Abstract])) OR (Cancer, Bladder[Title/Abstract])) OR (Cancer of Bladder[Title/Abstract])) OR (bladder cancer[Title/Abstract])	50349
2	HIVEC[Title/Abstract]	36
3	(#1) AND (#2)	31
4	((((CHT[Title/Abstract]) OR (chemohyperthermia[Title/Abstract])) OR (Thermo-chemotherapy[Title/Abstract])) OR device-assisted Therapy	2666
5	(((((((Therapy, Fever) OR (Hyperthermia, Therapeutic)) OR (Induced Hyperthermia)) OR (Therapeutic Hyperthermia)) OR (Thermotherapy)) OR (Fever Therapy)) OR (Hyperthermia, Local)) OR (Local Hyperthermia)	125804
6	((((((Therapy, Drug) OR (Drug Therapies)) OR (Therapies, Drug)) OR (Chemotherapy)) OR (Chemotherapies)) OR (Pharmacotherapy)) OR (Pharmacotherapies)	3890987
7	(#5) AND (#6)	65846
8	(#4) OR (#7)	68257
9	(#1) AND (#8)	452
10	(#3) OR (#9)	457
11	(#3) OR (#9) AND (randomizedcontrolledtrial[Filter])	31

1.2 Search terms in Ovid

All Ovid Journals (Abstracts Only)

Huazhong Univ Sci & Technol Full Text Journals(LWW+ADIS)

Embase <1974 to 2023 January 09>

Books@Ovid <December 27, 2022>

Ovid MEDLINE(R) ALL <1946 to January 09, 2023>

1 bladder cancer/ or bladder tumor/ or bladder carcinogenesis/ or bladder carcinoma/ or bladder metastasis/ or muscle invasive bladder cancer/ or non muscle invasive bladder cancer/ 162324
2 HIVEC.ab,kw,ti. 112
3 1 and 2 73
4 CHT.ab,kw,ti. 6211
5 chemohyperthermia.ab,kw,ti. 477
6 Thermo-chemotherapy.ab,kw,ti. 366
7 device-assisted therapy.ab,kw,ti. 68
8 4 or 5 or 6 or 77058
9 adjuvant chemotherapy/ or chemotherapy/ 292458
10 microwave thermotherapy/ or thermotherapy/ 30673
11 hyperthermia/ 24378
12 10 or 11 54711
13 9 and 12 1323
14 1 and 13 61
15 3 or 14 131
16 clinical trial/ or controlled clinical trial/ or randomized controlled trial/ or controlled study/ 10835564
17 15 and 1645

1.3 Search terms in Cochrane Library

#1 MeSH descriptor: [Urinary Bladder Neoplasms] explode all trees 1645
#2 HIVEC 19
#3 Hyperthermia Intravesical Chemotherapy 19
#4 #2 OR #3 33
#5 #1 AND #4 13
#6 CHT 254
#7 chemohyperthermia40
#8 Thermo-chemotherapy 8
#9 device-assisted therapy 33
#10 #6 OR #7 OR #8 OR #9 332
#11 MeSH descriptor: [Drug Therapy] explode all trees 148743
#12 MeSH descriptor: [Hyperthermia, Induced] explode all trees 1845
#13 #11 OR #12 300
#14 #10 OR #13 629
#15 #1 AND #14 15
#16 #5 OR #15 17
Filter：Trials - 15

Part 2: BCG versus MMC versus TURB

2.1 Search terms in PubMed

PubMed	Search terms	results

1	((((((((((((((((Neoplasm, Urinary Bladder[Title/Abstract]) OR (Urinary Bladder Neoplasm[Title/Abstract])) OR (Bladder Tumors[Title/Abstract])) OR (Bladder Tumor[Title/Abstract])) OR (Tumor, Bladder[Title/Abstract])) OR (Tumors, Bladder[Title/Abstract])) OR (Neoplasms, Bladder[Title/Abstract])) OR (Bladder Neoplasms[Title/Abstract])) OR (Bladder Neoplasm[Title/Abstract])) OR (Neoplasm, Bladder[Title/Abstract])) OR (Urinary Bladder Cancer[Title/Abstract])) OR (Cancer, Urinary Bladder[Title/Abstract])) OR (Malignant Tumor of Urinary Bladder[Title/Abstract])) OR (Cancer of the Bladder[Title/Abstract])) OR (Bladder Cancer[Title/Abstract])) OR (Bladder Cancers[Title/Abstract])) OR (Cancer, Bladder[Title/Abstract])) OR (Cancer of Bladder[Title/Abstract])) OR (bladder cancer[Title/Abstract])	50349
2	((((((((((mitomycin C[Title/Abstract]) OR (Mitomycin C[Title/Abstract])) OR (Mitomycin-C[Title/Abstract])) OR (Mitocin-C[Title/Abstract])) OR (Mitocin C[Title/Abstract])) OR (MitocinC[Title/Abstract])) OR (NSC-26980[Title/Abstract])) OR (NSC 26980[Title/Abstract])) OR (NSC26980[Title/Abstract])) OR (Ametycine[Title/Abstract])) OR (Mutamycin[Title/Abstract])) OR (MMC[Title/Abstract])	21120
3	(((((((BCG[Title/Abstract]) OR (Vaccine, BCG[Title/Abstract])) OR (Bacillus Calmette Guerin Vaccine[Title/Abstract])) OR (Calmette's Vaccine[Title/Abstract])) OR (Calmette Vaccine[Title/Abstract])) OR (Calmettes Vaccine[Title/Abstract])) OR (Vaccine, Calmette's[Title/Abstract])) OR (Calmette Guerin Bacillus Vaccine[Title/Abstract])	25881
4	(#2) OR (#3)	46672
5	(#1) AND (#4)	3732
6	(#1) AND (#4) AND (randomizedcontrolledtrial[Filter])	224

2.2 Search terms in Ovid

All Ovid Journals (Abstracts Only)

Huazhong Univ Sci & Technol Full Text Journals(LWW+ADIS)

Embase <1974 to 2023 January 09>

Books@Ovid <December 27, 2022>
Ovid MEDLINE(R) ALL <1946 to January 09, 2023>

1 bladder cancer/ or bladder tumor/ or bladder carcinogenesis/ or bladder carcinoma/ or bladder metastasis/ or muscle invasive bladder cancer/ or non muscle invasive bladder cancer/ 162324
2 mitomycin/ 36444
3 mycobacterium bovis bcg/ 3441
4 2 or 3 39712
5 1 and 4 4224
6 randomized controlled trial/ 1328772
7 5 and 6 328

2.3 Search terms in Cochrane Library

#1 MeSH descriptor: [Urinary Bladder Neoplasms] explode all trees 1645
#2 MeSH descriptor: [Mitomycin] explode all trees 1210
#3 MeSH descriptor: [Mycobacterium bovis] explode all trees 90
#4 #2 OR #3 1299
#5 #1 AND #4 187
Filter： Randomized Controlled Trial – 183
