# Supplementary material for: Hyperthermia intravesical chemotherapy acts as a promising alternative to bacillus Calmette–Guérin instillation in non-muscle-invasive bladder cancer: a network meta-analysis
Source: Front Oncol. 2023 May 12;13:1164932. doi: 10.3389/fonc.2023.1164932 (PMC10213538; doi:10.3389/fonc.2023.1164932)
Supplement: File S2 — the exact reasons for excluding 38 articles [file DataSheet_6.pdf]

Reasons for excluded articles

Author	Reasons for article exclusion
R. Colombo, et al.[1]	No more statistics are provided to obtain HR of RFS/PFS
A. Sousa, et al.[2]	It is a single-arm clinical trial
J. Kelly, et al.[3]	The setting of its control group is undesirable
M. Ba, et al.[4]	No more statistics are provided to obtain HR of RFS/PFS
Y. B. Thyavihally[5]	It is a retrospective study
M. T. Melgarejo Segura[6]	It is a unrandomized clinical trial
W. S. Tan, et al.[7]	No full text
D. A. Gonzalez Padilla, et al.[8]	No more statistics are provided to obtain HR of RFS/PFS
N. Rodrigues Netto, et al.[9]	No more statistics are provided to obtain HR of RFS/PFS
H. Huland and U. Otto[10]	No more statistics are provided to obtain HR of RFS/PFS
H. Huland, et al.[11]	No more statistics are provided to obtain HR of RFS/PFS
U. Maier and J. H. Holzner[12]	No more statistics are provided to obtain HR of RFS/PFS
F. M. DeBruyne, et al.[13]	No more statistics are provided to obtain HR of RFS/PFS
C. A. D'Ancona, et al.[14]	No more statistics are provided to obtain HR of RFS/PFS
N. R. Netto Júnior, et al.[15]	No more statistics are provided to obtain HR of RFS/PFS
T. Tsushima, et al.[16]	No more statistics are provided to obtain HR of RFS/PFS
D. L. Lamm, et al.[17]	No more statistics are provided to obtain HR of RFS/PFS
W. P. Witjes, et al.[18]	No more statistics are provided to obtain HR of RFS/PFS
C. Cheng, et al.[19]	No more statistics are provided to obtain HR of RFS/PFS
D. A. Tolley, et al.[20]	No more statistics are provided to obtain HR of RFS/PFS

W. P. Witjes, et al.[21]	No more statistics are provided to obtain HR of RFS/PFS
P. U. Malmström, et al.[22]	No more statistics are provided to obtain HR of RFS/PFS
E. Solsona, et al.[23]	No more statistics are provided to obtain HR of RFS/PFS
H. Wang, et al.[24]	No full text
J. Palou, et al.[25]	No more statistics are provided to obtain HR of RFS/PFS
A. Kolodziej, et al.[26]	No more statistics are provided to obtain HR of RFS/PFS
M. D. Shelley, et al.[27]	It is a review
M. Tong, et al.[28]	No full text
S. El-Ghobashy, et al.[29]	No full text
B. Mangiarotti, et al.[30]	No full text
A. L. Sabichi, S, et al.[31]	The setting of trial groups is undesirable
F. Wang, et al.[32]	No full text
F. Kunieda, et al.[33]	The setting of its control group is undesirable
C. Verri, et al.[34]	No full text
H. P. Mondal, et al.[35]	No more statistics are provided to obtain HR of RFS/PFS
S. Di Stasi, et al.[36]	No more statistics are provided to obtain HR of RFS/PFS
D. Carrion Monsalve, et al.[37]	No more statistics are provided to obtain HR of RFS/PFS
B. Santosh, et al.[38]	No full text

1. Colombo, R., et al., *Neoadjuvant combined microwave induced local hyperthermia and topical chemotherapy versus chemotherapy alone for superficial bladder cancer.* J Urol, 1996. **155**(4): p. 1227-32.

2. Sousa, A., et al., *A clinical trial of neoadjuvant hyperthermic intravesical chemotherapy (HIVEC) for treating intermediate and high-risk non-muscle invasive bladder cancer.* International Journal of Hyperthermia, 2014. **30**(3): p. 166-70.

3. Kelly, J., et al., *HYMN: A randomised controlled phase III trial comparing hyperthermia plus mitomycin to a second course of BCG or institutional standard in patients with recurrence of non-muscle invasive bladder cancer (NMIBC) following induction or maintenance BCG therapy.* BJU International, 2015. **115(Supplement 7)**: p. 12.

4. Ba, M., et al., *Bladder intracavitary hyperthermic perfusion chemotherapy for the prevention of recurrence of non-muscle invasive bladder cancer after transurethral resection.* Oncol Rep, 2017. **37**(5): p. 2761-2770.

5. Thyavihally, Y.B., et al., *Comparing adverse effects, short term outcomes, and cost implications of hyperthermic intravesical chemotherapy with Mitomycin-C and intravesical bacillus Calmette-Guerin instillation (Moscow-I strain) in the management of intermediate and high-risk nonmuscle invasive bladder cancer.* Urol Ann, 2021. **13**(4): p. 424-430.

6. Melgarejo Segura, M.T., et al., *Conductive hyperthermic chemotherapy versus electromotive drug administration of mitomycin C as intravesical adjuvant treatment of patients with intermediate or high-risk non-muscle invasive bladder cancer.* Urologic Oncology: Seminars and Original Investigations., 2022.

7. Tan, W.S., J. Palou, and J. Kelly, *Safety and tolerability analysis of hyperthermic intravesical mitomycin to mitomycin alone in HIVEC I and HIVEC II: An interim analysis of 307 patients.* European Urology, Supplements, 2017. **16(3)**: p. e1150-e1151.

8. Gonzalez Padilla, D.A., et al., *HIVEC HR: Chemohyperthermia with mitomycin C vs BCG for high-risk non-muscle invasive bladder cancer. Preliminary results from a randomized controlled trial.* European Urology, Supplements, 2019. **18(1)**: p. e768-e770.

9. Rodrigues Netto, N., Jr., G. Caserta Lemos, and N. de Belis, *[The BCG as coadjuvant in the treatment of bladder carcinoma].* Arch Esp Urol, 1979. **32**(5): p. 417-26.

10. Huland, H. and U. Otto, *Mitomycin instillation to prevent recurrence of superficial bladder carcinoma. Results of a controlled, prospective study in 58 patients.* European Urology, 1983. **9(2)**: p. 84-86.

11. Huland, H., et al., *Long-term mitomycin C instillation after transurethral resection of superficial bladder carcinoma: influence on recurrence, progression and survival.* J Urol, 1984. **132**(1): p. 27-9.

12. Maier, U. and J.H. Holzner, *The value of urothelial dysplasias in normal looking bladder mucosa from patients with bladder tumors. Results of a prospective study with and without prophylactic intravesical chemotherapy with mitomycin C. [German].* Onkologie, 1985. **8(4)**: p. 232-240.

13. DeBruyne, F.M., et al., *BCG (RIVM) versus mitomycin intravesical therapy in superficial bladder cancer. First results of randomized prospective trial.* Urology, 1988. **31**(3 Suppl): p. 20-5.

14. D'Ancona, C.A., et al., *Oral or intravesical bacillus Calmette-Guerin immunoprophylaxis in bladder carcinoma.* J Urol, 1991. **145**(3): p. 498-501.

15. Netto Júnior, N.R., et al., *Immunoprophylaxis of superficial bladder cancer: a prospective and randomized comparison of oral versus intravesical Bacillus Calmette-Guerin.* Arch Esp Urol, 1991. **44**(8): p. 1025-8.

16. Tsushima, T., et al., *[Prophylactic intravesical instillation therapy in patients with superficial bladder cancer--results of a randomized prospective study].* Nihon Hinyokika Gakkai Zasshi, 1992. **83**(8): p. 1314-21.

17. Lamm, D.L., et al., *BCG immunotherapy of bladder cancer: inhibition of tumor recurrence and associated immune responses.* Cancer, 1981. **48**(1): p. 82-8.

18. Witjes, W.P., et al., *BCG-RIVM versus BCG-Tice versus mitomycin-C in superficial bladder cancer. Rationale, design, and interim analysis of the trial of the South-East Cooperative Urological Group, The Netherlands.* Prog Clin Biol Res, 1992. **378**: p. 59-67.

19. Cheng, C., et al., *Bacillus Calmette-Guerin (BCG) in the treatment of superficial bladder cancer.* Ann Acad Med Singap, 1995. **24**(4): p. 562-5.

20. Tolley, D.A., et al., *The effect of intravesical mitomycin C on recurrence of newly diagnosed superficial bladder cancer: a further report with 7 years of follow up.* J Urol, 1996. **155**(4): p. 1233-8.

21. Witjes, W.P., et al., *Update on the Dutch Cooperative Trial: mitomycin versus bacillus Calmette-Guérin-Tice versus bacillus Calmette-Guérin RIVM in the treatment of patients with pTA-pT1 papillary carcinoma and carcinoma in situ of the urinary bladder. Dutch South East Cooperative Urological Group.* Semin Urol Oncol, 1996. **14**(1 Suppl 1): p. 10-6.

22. Malmström, P.U., et al., *5-year followup of a randomized prospective study comparing mitomycin C and bacillus Calmette-Guerin in patients with superficial bladder carcinoma. Swedish-Norwegian Bladder Cancer Study Group.* J Urol, 1999. **161**(4): p. 1124-7.

23. Solsona, E., et al., *Effectiveness of a single immediate mitomycin C instillation in patients with low risk superficial bladder cancer: short and long-term followup.* J Urol, 1999. **161**(4): p. 1120-3.

24. Wang, H., et al., *Prevention and treatment of bladder tumors recurrence with furtulon. [Chinese].* Zhongguo yi xue ke xue yuan xue bao, 2000. **Acta Academiae Medicinae Sinicae. 22(1)**: p. 79-81.

25. Palou, J., et al., *Control group and maintenance treatment with bacillus Calmette-Guerin for carcinoma in situ and/or high grade bladder tumors.* Journal of Urology, 2001. **165(5 I)**: p. 1488-1491.

26. Kolodziej, A., et al., *Treatment of high-risk superficial bladder cancer with maintenance bacille Calmette-Guérin therapy: preliminary results.* BJU Int, 2002. **89**(6): p. 620-2.

27. Shelley, M.D., et al., *Intravesical bacillus Calmette-Guerin versus mitomycin C for Ta and T1 bladder cancer.* Cochrane database of systematic reviews (Online), 2003(3): p. CD003231.

28. Tong, M., et al., *Prevention of postoperative recurrence of human bladder carcinoma by intravesical instillation of immunotoxin, a clinical study. [Chinese].* Zhonghua yi xue za zhi, 2003. **83(3)**: p. 201-203.

29. El-Ghobashy, S., et al., *Effectiveness of a single immediate mitomycin C instillation in patients with low risk superficial bladder cancer: short and long-term follow-up.* J Egypt Natl Canc Inst, 2007. **19**(2): p. 121-6.

30. Mangiarotti, B., et al., *A randomized prospective study of intravesical prophylaxis in non-musle invasive bladder cancer at intermediate risk of recurrence: mitomycin chemotherapy vs BCG immunotherapy.* Arch Ital Urol Androl, 2008. **80**(4): p. 167-71.

31. Sabichi, A.L., et al., *Phase III prevention trial of fenretinide in patients with resected non-muscle-invasive bladder cancer.* Clin Cancer Res, 2008. **14**(1): p. 224-9.

32. Wang, F., et al., *Prospective clinical studies at the efficacy of brucea javanica oil, mitomycin and BCG for preventing postoperative relapse of superficial bladder cancer through perfusion.* Chinese-German Journal of Clinical Oncology, 2011. **10(4)**: p. 228-231.

33. Kunieda, F., et al., *Watchful waiting versus intravesical BCG therapy for high-grade pT1 bladder cancer with pT0 histology after second transurethral resection: Japan Clinical Oncology Group Study JCOG1019.* Jpn J Clin Oncol, 2012. **42**(11): p. 1094-8.

34. Verri, C., et al., *Intravesical adjuvant electromotive mitomycin-C in patients with primary intermediate-risk non-muscle invasive bladder cancer: A randomized controlled trial.* Anticancer Research, 2012. **32(5)**: p. 1860-1861.

35. Mondal, H.P., et al., *Prospective randomized study between intravesical BCG and mitomycin-C for non-muscleinvasive urothelial carcinoma of urinary-bladder post TURBT.* Bangladesh Journal of Medical Science, 2016. **15(1)**: p. 74-77.

36. Di Stasi, S., et al., *Intravesical adjuvant electromotive mitomycin-C in patients with primary intermediate-risk urothelial non-muscle invasive bladder cancer: A randomized controlled trial.* European Urology, Supplements, 2019. **18(9)**: p. e3248.

37. Carrion Monsalve, D., et al., *The effect of a neoadjuvant instillation of chemotherapy in the prevention of recurrences of non-muscle invasive bladder cancer. Preliminary results of the PRECAVE prospective, controlled, randomized clinical trial.* European Urology, 2021. **79(Supplement 1)**: p. S1043-S1044.

38. Santosh, B., et al., *Comparison of Efficacy and Safety of Intravesical Therapy for Non-Muscle Invasive Bladder Cancer with Intravesical Bacillus Calmette-Guerin Vs Mitomycin C: A Prospectice Randomised Syudy.* International Journal of Academic Medicine and Pharmacy, 2022. **4(3)**: p. 256-260.
