# Supplementary material for: Hyperthermia intravesical chemotherapy acts as a promising alternative to bacillus Calmette–Guérin instillation in non-muscle-invasive bladder cancer: a network meta-analysis
Source: Front Oncol. 2023 May 12;13:1164932. doi: 10.3389/fonc.2023.1164932 (PMC10213538; doi:10.3389/fonc.2023.1164932)

# A. Risk of bias figures

| Unique ID | Experimental | Comparator | Outcome | Weight | Randomization process | Deviations from intended interv | Missing outcome data | Measurement of the outcome | Selection of the reported result | Overall |
|---|---|---|---|---|---|---|---|---|---|---|
| 1-Tom2016 | HIVEC | BCG | RFS | 1 | + | ? | + | + | + | ! |
| 1-Wei2019 | HIVEC | BCG | RFS | 1 | + | + | + | + | + | + |
| 1-Guerrero2023 | HIVEC | BCG | RFS, PFS | 1 | + | + | + | + | + | + |
| 2-Colombo2010 | HIVEC | MMC | RFS | 1 | + | + | + | + | + | + |
| 2-Wei2023 | HIVEC | MMC | RFS, PFS | 1 | + | + | + | + | + | + |
| 3-Rintala1991 | BCG | MMC | RFS | 1 | + | ? | + | + | + | ! |
| 3-J. A.1998 | BCG | MMC | RFS, PFS | 1 | + | + | + | + | + | + |
| 3-Friedrich2007 | BCG | MMC | RFS | 1 | + | + | + | + | + | + |
| 3-Gårdmark2007 | BCG | MMC | PFS | 1 | + | + | + | + | + | + |
| 3-Ojea2007 | BCG | MMC | RFS, PFS | 1 | + | + | + | + | + | + |
| 3-Isbarn2008 | BCG | MMC | RFS | 1 | + | + | + | + | + | + |
| 5-Niijima1983 | MMC | TURB | RFS | 1 | + | + | + | + | + | + |
| 5-Tsushima1987 | MMC | TURB | RFS | 1 | + | + | + | + | + | + |
| 4-Yamamoto1990 | BCG | TURB | RFS | 1 | + | + | + | + | + | + |
| 3-S. KREGE1996 | BCG, MMC | TURB | RFS | 1 | + | + | + | + | + | + |
| 5-SAKAMOTO2001 | MMC | TURB | RFS | 1 | + | + | + | + | + | + |
| 5-De Nunzio2011 | MMC | TURB | RFS | 1 | + | + | + | + | + | + |
| 5-Akaza1987 | MMC | TURB | RFS | 1 | + | + | + | + | + | + |
| 5-HYEON1989 | MMC | TURB | RFS, PFS | 1 | + | + | + | + | + | + |
| 4-FRANCESCO1991 | BCG | TURB | RFS, PFS | 1 | + | + | + | + | + | + |
| 4-Melekos1993 | BCG | TURB | RFS, PFS | 1 | + | + | + | + | + | + |
| 3-PETER1995 | BCG1,2 | MMC | RFS, PFS | 1 | + | + | + | + | + | + |
| 4-Harry1995 | BCG | TURB | RFS, PFS | 1 | + | ? | + | + | + | ? |
| 3-Donald1995 | BCG | MMC | RFS, PFS | 1 | + | + | + | + | + | + |
| 5-TOLLEY1996 | MMC | TURB | RFS, PFS | 1 | + | + | + | + | + | + |
| 3-PER-UNO1999 | BCG | MMC | RFS | 1 | + | + | + | + | + | + |
| 3-Barbara2008 | BCG | MMC | RFS | 1 | + | + | + | + | + | + |
| 3-Riikka2009 | BCG | MMC | RFS, PFS | 1 | + | + | + | + | + | + |
| 2-Angulo2023 | HIVEC | MMC | RFS | 1 | + | + | + | + | + | + |
| 4-Herr1997 | BCG | TURB | PFS | 1 | + | + | + | + | + | + |
| 4-lamm1980 | MMC | TURB | RFS | 1 | + | + | + | + | + | + |
| 3-J.A1993 | BCG | MMC | RFS | 1 | + | + | + | + | + | + |
| 3-lundholm1996 | BCG | MMC | RFS | 1 | + | + | + | + | + | + |
| 3-Geert1998 | BCG | MMC | RFS, PFS | 1 | + | + | + | + | + | + |

+ Low risk
? Some concerns
? High risk

# B. Risk of bias graph

| | Randomization process | Deviations from intended interventions | Mising outcome data | Measurement of the outcome | Selection of the reported result | Overall Bias |
|---|---|---|---|---|---|---|
| Total number of study = 34 | | | | | | |
| Low risk | 100 | 91.2 | 100 | 100 | 100 | 91.2 |
| Some concerns | 0 | 5.9 | 0 | 0 | 0 | 5.9 |
| High risk | 0 | 2.9 | 0 | 0 | 0 | 2.9 |

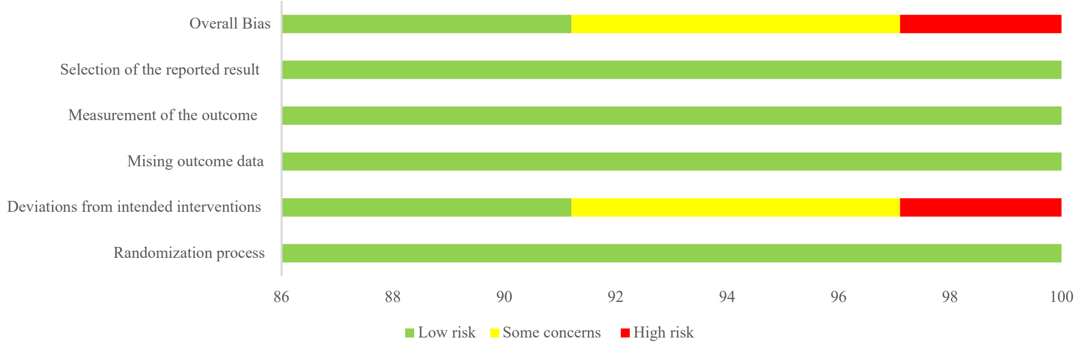

Supplement: Supplementary Figure 1 — the quality assessment figures and graph using RoB2 [file DataSheet_1.pdf]
