# Supplementary figures and images for: Hyperthermia intravesical chemotherapy acts as a promising alternative to bacillus Calmette–Guérin instillation in non-muscle-invasive bladder cancer: a network meta-analysis
Source: Front Oncol. 2023 May 12;13:1164932. doi: 10.3389/fonc.2023.1164932 (PMC10213538; doi:10.3389/fonc.2023.1164932)

A. Recurrence

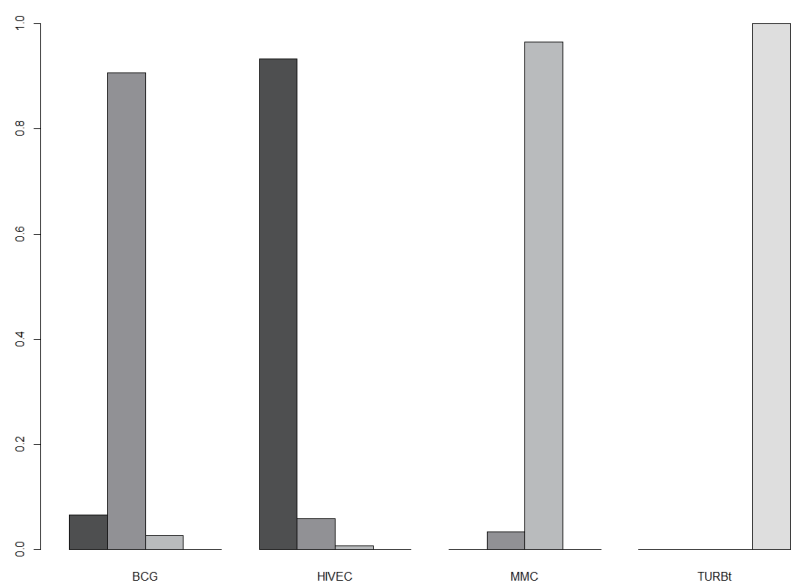

B. Progression

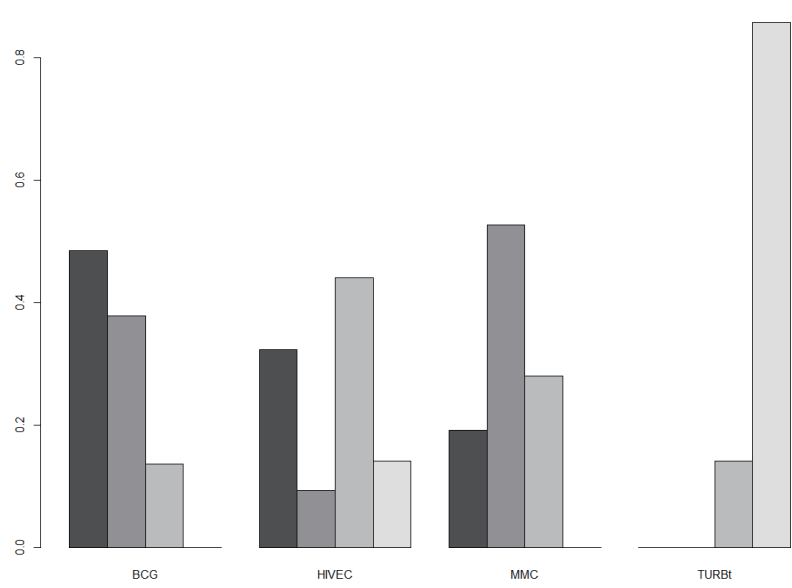

Supplement: Supplementary Figure 2 — posterior ranking probabilities of four treatment strategies for recurrence and progression [file DataSheet_2.pdf]

# A. Consistency check for HR of RFS

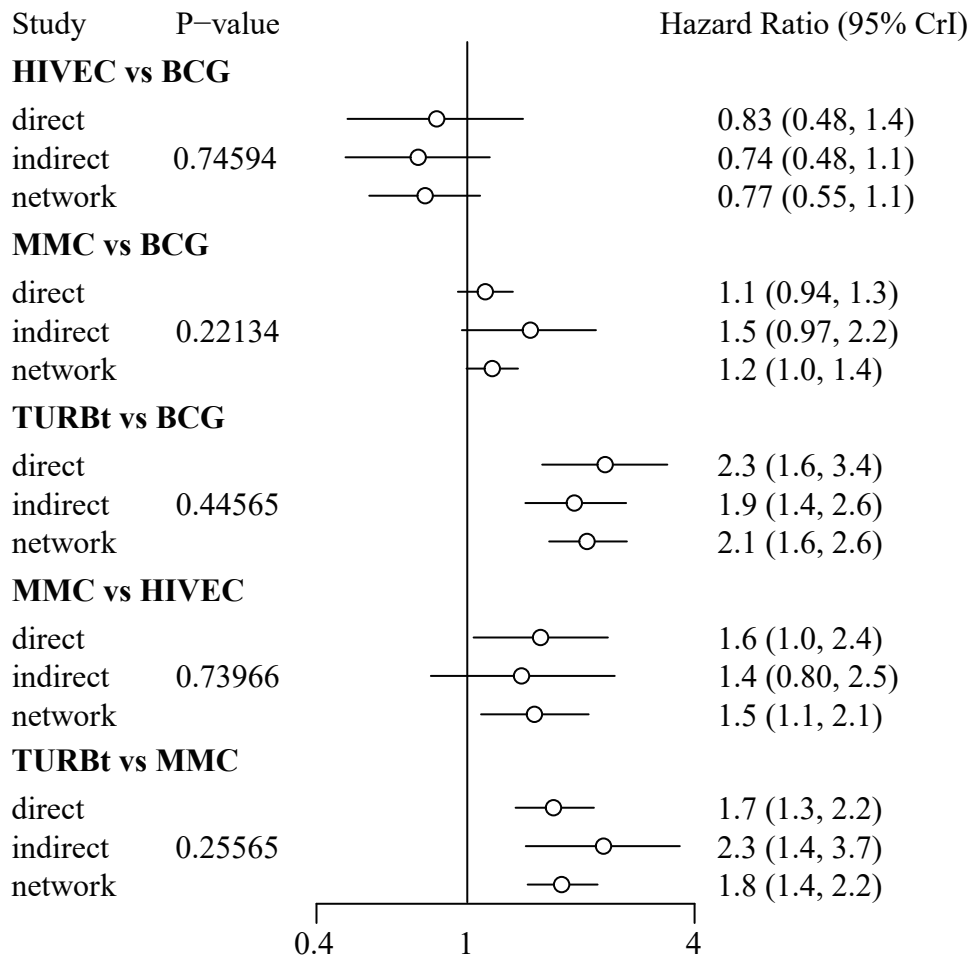

# B. Consistency check for HR of PFS

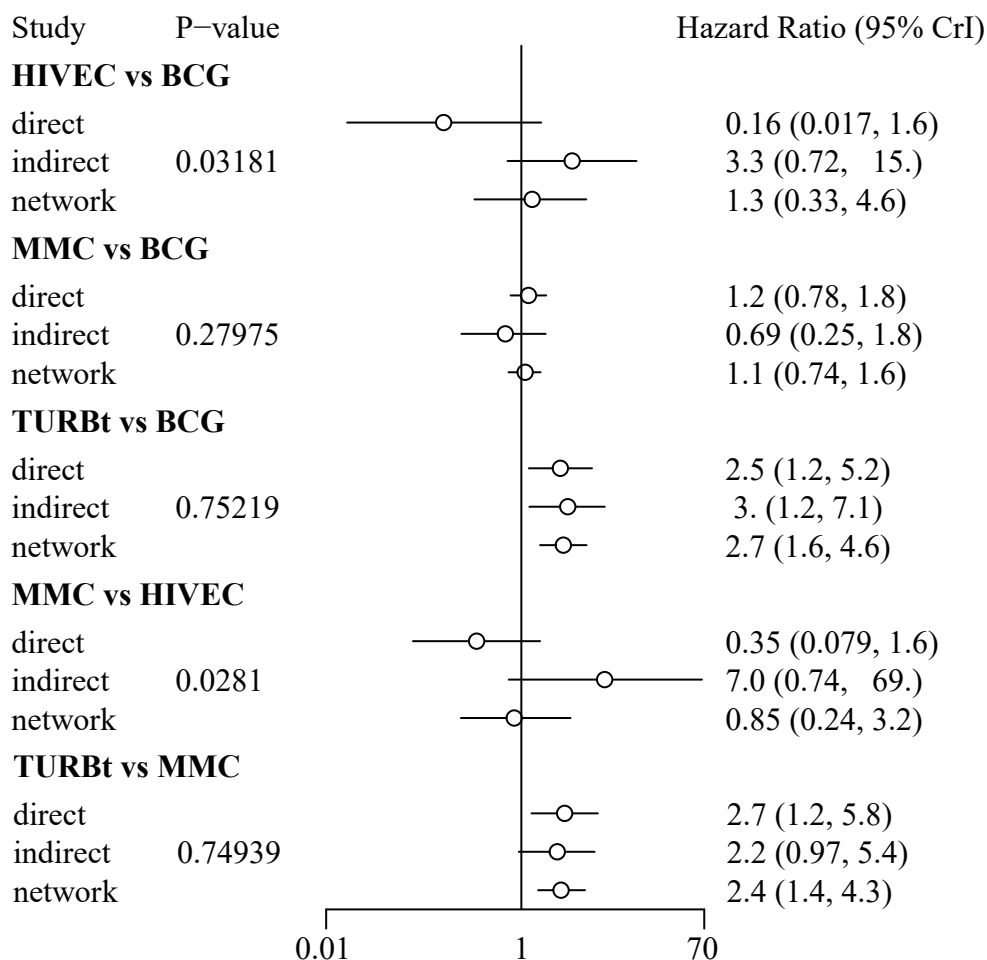

Supplement: Supplementary Figure 3 — results of consistency check [file DataSheet_3.pdf]

## A. Heterogeneity test for HR of RFS

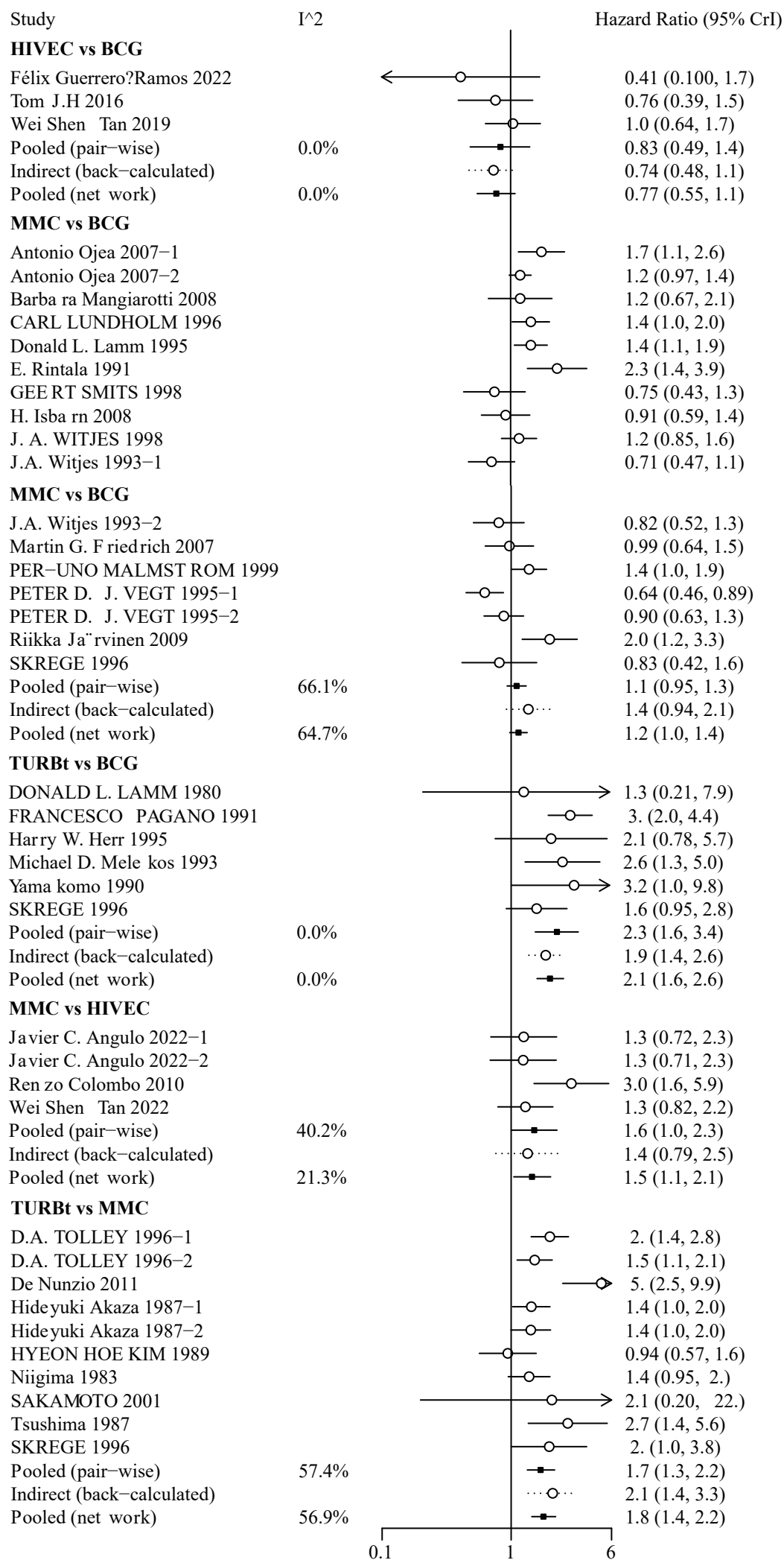

## B. Heterogeneity test for HR of PFS

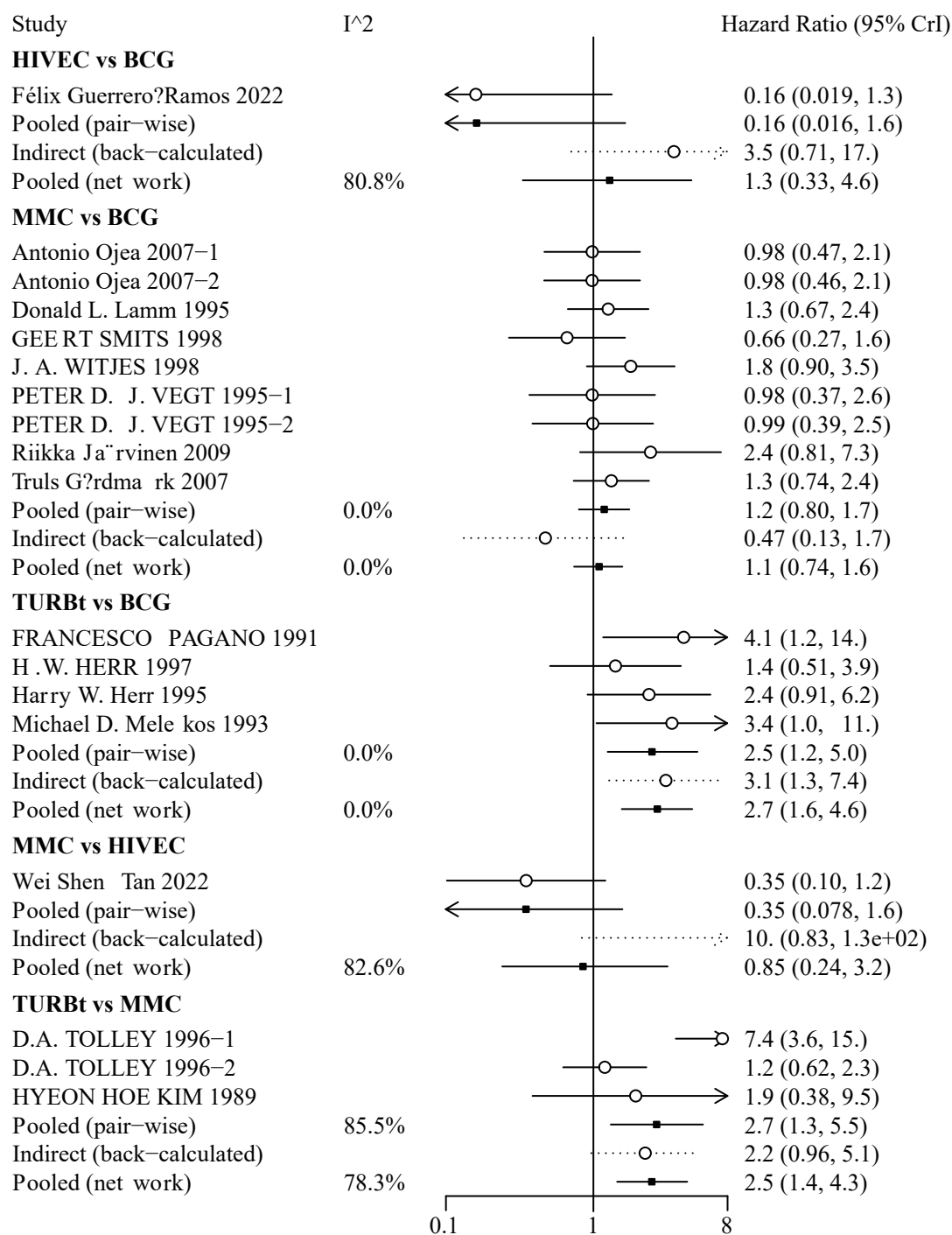

Supplement: Supplementary Figure 4 — results of heterogeneity test [file DataSheet_4.pdf]
